# Genomic diversity of human-associated *Mycobacterium bovis* in Canada

**DOI:** 10.1128/spectrum.01485-26

**Published:** 2026-10-13

**Authors:** Meenu K. Sharma, Olga Andrievskaia, Gregory J. Tyrrell, Pierre-Marie Akochy, Inna Sekirov, Melissa Richard-Greenblatt, Angela Ma, Heather Adam, Mohey Alawa, Elizabeth Simms, Md Rashedul Islam, Melissa Rabb, Hafid Soualhine

**Affiliations:** 1 National Reference Centre for Mycobacteriology, National Microbiology Laboratory, Public Health Agency of Canada 41687, Winnipeg, Manitoba, Canada; 2 Medical Microbiology and Infectious Diseases, University of Manitoba 8664, Winnipeg, Canada; 3 National Reference Laboratory for Bovine Tuberculosis, Ottawa Animal Health Laboratory, Canadian Food Inspection Agency 5737, Ottawa, Ontario, Canada; 4 Division of Diagnostic and Applied Microbiology, Laboratory Medicine and Pathology, University of Alberta and Alberta Precision Laboratories, Edmonton, Alberta, Canada; 5 Laboratoire de santé publique du Québec, INSPQ 54470 , Québec City, Canada; 6 Public Health Laboratory, BC Centre for Disease Control 113269, Vancouver, British Columbia, Canada; 7 Public Health Ontario 153300, Toronto, Ontario, Canada; 8 Laboratory Medicine and Pathobiology, University of Toronto 7938, Toronto, Canada; 9 Department of Pediatric Laboratory Medicine, The Hospital for Sick Children 7979, Toronto, Canada; 10 Shared Health, Health Sciences Centre 103093, Winnipeg, Manitoba, Canada; 11 Regina Qu'Appelle Health Region 7234, Regina, Saskatchewan, Canada; 12 Provincial Public Health Laboratory, Nova Scotia, Canada; 13 McGill International TB Research Centre, McGill University 5620, Montreal, Quebec, Canada; Taichung Veterans General Hospital, Taichung, Taiwan

**Keywords:** *Mycobacterium bovis*, genomic relatedness, antimicrobial resistance, bovine tuberculosis

## Abstract

Human infections caused by *Mycobacterium bovis* in Canada are not well documented. The following study analyzed *M. bovis* (excluding BCG) isolates identified in public health laboratories across Canadian provinces between 2000 and 2025. Whole-genome sequencing was used to catalog mutations linked to antimicrobial resistance and evaluate the genomic relatedness of isolates. The majority of samples in this study were non-respiratory (60%). Of 74 study isolates, 69 (93%) were phenotypically sensitive to first-line drugs isoniazid, rifampin, and ethambutol. The most frequently encountered *M. bovis* clusters belonged to *M. tuberculosis* complex lineages La 1.7 and La 1.8. Although some isolates showed phylogenetic clustering, their lack of spatial and temporal relatedness suggested sporadic cases rather than local *M. bovis* transmission. Of all first-line drugs evaluated, isoniazid showed the lowest concordance (94.4%) between phenotypic and genotypic methods. The isolates in this study harbor some mutations that are not currently documented in the WHO mutation catalog, indicating that these variants may be rare. The findings suggest that the prevalence of *M. bovis* infection in Canada is low with cases unlikely to be acquired through human-to-human transmission.

**IMPORTANCE:**

Incidence of human infections due to *Mycobacterium bovis* in Canada is largely unknown as these data are not collected or annually published. This research is a first effort to identify historical *M. bovis* isolates that were available for culture, had drug susceptibility testing data, and were analyzed through whole-genome sequencing. The annual reporting of these data in Canada holds value.

## INTRODUCTION

*Mycobacterium bovis* primarily infects animals, particularly cattle, although it has been occasionally observed in humans (1). *M. bovis* and its vaccine BCG strains (*M. bovis* Bacillus Calmette-Guerin) are classified within the *Mycobacterium tuberculosis* complex (MTBC). The species was first published in 1970 (2), added to the List of Prokaryotic names with Standing in Nomenclature in 1980 (3, 4), and later classified into several animal-adapted MTBC La1 sublineages (5). *M. bovis* can thrive within the host for extended durations, successfully establishing chronic infections after a lengthy incubation period (2, 6). It results in a considerable disease burden, along with financial and economic repercussions in endemic regions (7–12). Occupational exposure to *M. bovis* continues to be a considerable concern for humans (13, 14) and requires treatment (15). First-line drugs used to treat *M. bovis* infection are the same as those used for *M. tuberculosis* treatment, including isoniazid, rifampin, and ethambutol, except for pyrazinamide, to which *M. bovis* is intrinsically resistant. To improve management guidelines for *M. bovis*, it is imperative to collect robust epidemiological data and treatment outcomes.

There is under-reporting of zoonotic tuberculosis (zTB) globally, and strengthening of human and zoonotic surveillance systems is needed to better understand their impact, emphasizing a One-Health approach (16–18). In 2019, it was estimated that there were approximately 140,000 new human cases of zTB worldwide (a range of 69,800–235,000) (17), and the estimated global incidence of tuberculosis (TB) was 10 million (a range of 8.9–11.0 million) with 1.2 million TB-related deaths among HIV-negative individuals and 208,000 deaths among HIV-positive cases. According to the World Health Organization (WHO), the global incident cases of people becoming ill with TB disease have remained above 10 million annually since 2010 (19, 20); however, current data for zoonotic disease are not well documented.

Diagnostic testing for *M. bovis* for human samples follows the same standards as other members of MTBC such as acid-fast bacilli smear and culture, identification, drug susceptibility testing (DST), and genotyping (21, 22). Genomic data analysis plays a crucial role in assessing infection transmission, as it provides comprehensive information at the base pair level, supplemented by single-nucleotide variants (SNV) matrices. The implementation of bioinformatics pipelines and workflows for deciphering transmission, identification, and molecular DST enhances the efficiency of data analysis; however, the standardization of these tools remains a significant challenge.

The objective of this study was to understand the geographic distribution, phylogenetic relatedness, and drug resistance patterns of all human- associated *M. bovis* isolates, excluding BCG, available at the National Reference Centre for Mycobacteriology (NRCM) and Canadian provincial mycobacteriology laboratories.

## MATERIALS AND METHODS

### Sample selection

All available human *M. bov*is isolates identified through routine mycobacterial culture methods at Canadian provincial laboratories (Alberta Precision Laboratories, Laboratoire de santé publique du Québec, BC Centre for Disease Control—British Columbia, Public Health Ontario, Shared Health: Health Sciences Centre—Manitoba, Regina Qu'Appelle Health Region—Saskatchewan, Provincial Public Health Laboratory—Nova Scotia) between 2000 and 2025 were submitted to the NRCM Laboratory (Winnipeg, Canada) or to the National Reference Laboratory for Bovine Tuberculosis (NRLbTb), Canadian Food Inspection Agency, and were included in this study. This number is not reflective of the total *M. bovis* identified in Canada, and the total number is likely higher.

### MTBC identification and phenotypic drug susceptibility testing

Smear, culture, and phenotypic DST were performed either by provincial laboratories or by the NRCM as previously described (23, 24). The samples were decontaminated using NALC-NaOH methodology, and the resulting pellet was inoculated into MGIT 960 vials with OADC growth supplement for mycobacterial growth (24). Phenotypic DST was performed at a critical concentration of four first line anti-tuberculosis drugs using BACTEC 960 MGIT (Becton Dickinson, Mississauga, Ontario, Canada) methodology with *M. tuberculosis* H37Rv ATCC 27294 strain run in parallel to all tests (24–28). Critical concentrations for isoniazid, rifampin, ethambutol, and pyrazinamide were 0.1 μg/mL, 10 μg/mL, 5.0 μg/mL, and 100 μg/mL, respectively. Pyrazinamide testing was conducted utilizing standard MTBC DST testing, as the species identification of the isolate was not known at the time of DST testing. For this study, whole-genome sequencing (WGS) data were used to identify mycobacterial species as described below.

### Whole-genome sequencing and data analyses

A 10 μL loopful of pure bacterial culture from Middlebrook 7H10 media (Sigma-Aldrich, ON, Canada) or 2 mL of liquid culture from BACTEC 960 MGIT (BACTEC, Mississauga, ON, Canada) was used for genomic DNA extraction of all clinical samples. An additional four control strains were used due to their historical significance: *M. bovis* ATCC 19210 (Karlson and Lessel Type strain), *M. bovis* ATCC 35720 (Ravenel, isolated before 1910), *M. bovis* BCG ATCC 35732 (vaccine BCG Sweden), and *M. bovis* BCG ATCC 35733 (vaccine BCG Danish) (ATCC, Manassas, VA, USA) (2, 29). DNA extraction was performed using the DNeasy UltraClean Microbial Kit (Qiagen, Maryland, USA). DNA libraries were prepared with the Illumina DNA Prep kit (Illumina, San Diego, CA, USA) and sequenced on the MiSeq platform with a MiSeq Reagent Kit v2 or v3 (500-cycle; Illumina, San Diego, CA, USA), as previously described (23, 30).

Paired-end raw reads were subjected to quality control using the sequence quality workflow utilizing MultiQC (31), implemented in Galaxy v22.1 (32). BioHansel, a k-mers-based pipeline comprising positive and negative k-mers specific to members of the MTBC including *M. bovis* (33), was utilized for species identification as well as MTBC lineage determination. The Galaxy implementation of TB-Profiler v6.6.5 was used to analyze WGS data from clinical MTBC isolates for prediction of AMR to first-line drugs (34, 35).

Variant calling and pairwise comparison of SNVs between samples were determined by the Single-Nucleotide Variant PHYLogenomics (SNVPhyl) pipeline v.1.2.3 (36) within the Integrated Rapid Infectious Disease Analysis (IRIDA) Platform (37). Genomes with a minimum average base pair coverage of 40× were included in the analysis. Variant calling parameters were 90% minimum percent identity for a repeat region, a minimum mapped reads percent coverage of 80%, an SNV abundance ratio (alternative allele fraction) of 0.75, a minimum mean mapping quality of reads of a variant call of 30 for inclusion, and a minimum read coverage of 10 for identification of variants. High-density SNV regions were identified using a 200-bp window size with a threshold of ≥2 SNVs within the defined window size flagged as high density. Trimmed reads were aligned to the reference genome *M. bovis* AF2122/97 (GenBank accession number LT708304), which is 4,345,492 bp in length with 3,952 genes encoding proteins (38). The phylogenetic tree and SNV matrix generated from SNVPhyl were used to determine relatedness between isolates. To assess potential relatedness, isolates with a SNV difference of 0–25 were selected, and inference was made based on public health information, i.e., number of SNVs, year of infection, type of sample, and provincial residence of the patient. Isolates with ≤6 were considered related, and anything with more than 6 SNVs was investigated (39, 40).

## RESULTS

Over the study period (2000–2025), a total of 74 clinical human isolates tested at the NRCM were identified as *M. bovis* across the seven participating provinces and were, therefore, included in this study. The geographic distribution of isolates across the provinces were Quebec (*n* =19; 25.68%), Alberta (*n* = 19; 25.68%), British Columbia (*n* = 16; 21.62%), Ontario (*n* = 14; 18.92%), Manitoba (*n* = 4; 5.4%), Saskatchewan (*n* = 1; 1.35%), and Nova Scotia (*n* = 1; 1.35%). The annual distribution of clinical *M. bovis* isolates available from each province is shown in Table 1.

**TABLE 1:** Distribution of available *M. bovis* human clinical isolates across Canadian provinces, categorized by the year of culture submission and originating province^*a*^

Year received	Total isolates	BC	ON	NS	QC	AB	MB	SK
2000	1				1			
2001	1			1				
2003	2	1				1		
2004	2					1	1	
2005	1	1						
2007	1	1						
2008	2	2						
2009	3	1				2		
2011	4		2			2		
2012	2					1		1
2013	2		1			1		
2014	2		1			1		
2015	5	1	1			3		
2016	3		1		2			
2017	8		3		4	1		
2018	4	2	1		1			
2019	10	3	3		4			
2020	3	1				2		
2021	6	2	1		1	2		
2022	3	1			1		1	
2023	4				2	1	1	
2024	1				1			
2025	4				2	1	1	
TOTAL	74	16	14	1	19	19	4	1

^*a*^ BC, British Columbia; ON, Ontario; NS, Nova Scotia; QC, Quebec; AB, Alberta; MB, Manitoba; SK, Saskatchewan.

The estimated patient age range at the time of specimen collection varied from under 1 year to 90 years. There was 1 case for the age range of less than 1 year, 5 cases for 1–20 years, 32 cases for 21–40, 10 cases for 41–60, 11 cases over 60 years, and 15 were unknown.

The distribution of samples was categorized broadly into respiratory (21.6%; 16/74) and non-respiratory (59.5%; 44/74) and further stratified by specimen type as shown in Table 2. The source was unknown for 18.9% (14/74) of isolates. Out of the 44 non-respiratory samples, 29 (65.9%) were categorized as tissues, and 9 (20.5%) were aspirates. For 16 respiratory samples, 9 (56.3%) samples were isolated from sputum and followed by respiratory aspirates (3, 18.8%), respiratory tissues (2, 12.5%), and bronchial alveolar lavage (2, 12.5%).

**TABLE 2:** Distribution of samples by specimen source (respiratory vs non-respiratory) for human clinical *M. bovis* isolates collected across seven Canadian provinces, 2000–2025^*a*^

Sample source (number; %)	Specimen type	Count
Respiratory	Sputum	9
(*N* = 16; 21.6%)	Aspirate	3
	Tissue	2
	BAL	2
Non-respiratory	Aspirate	9
(*N* = 44; 59.5%)	Tissue	29
	Blood	1
	Urine	3
	Cerebral spinal fluid	1
	Stool	1
Unknown^*b*^	Unknown	14
(*N* = 14; 18.9%)		

^*a*^ BAL, bronchoalveolar lavage.

^*b*^ Data on the specimen source were not available.

**Fig 1:**
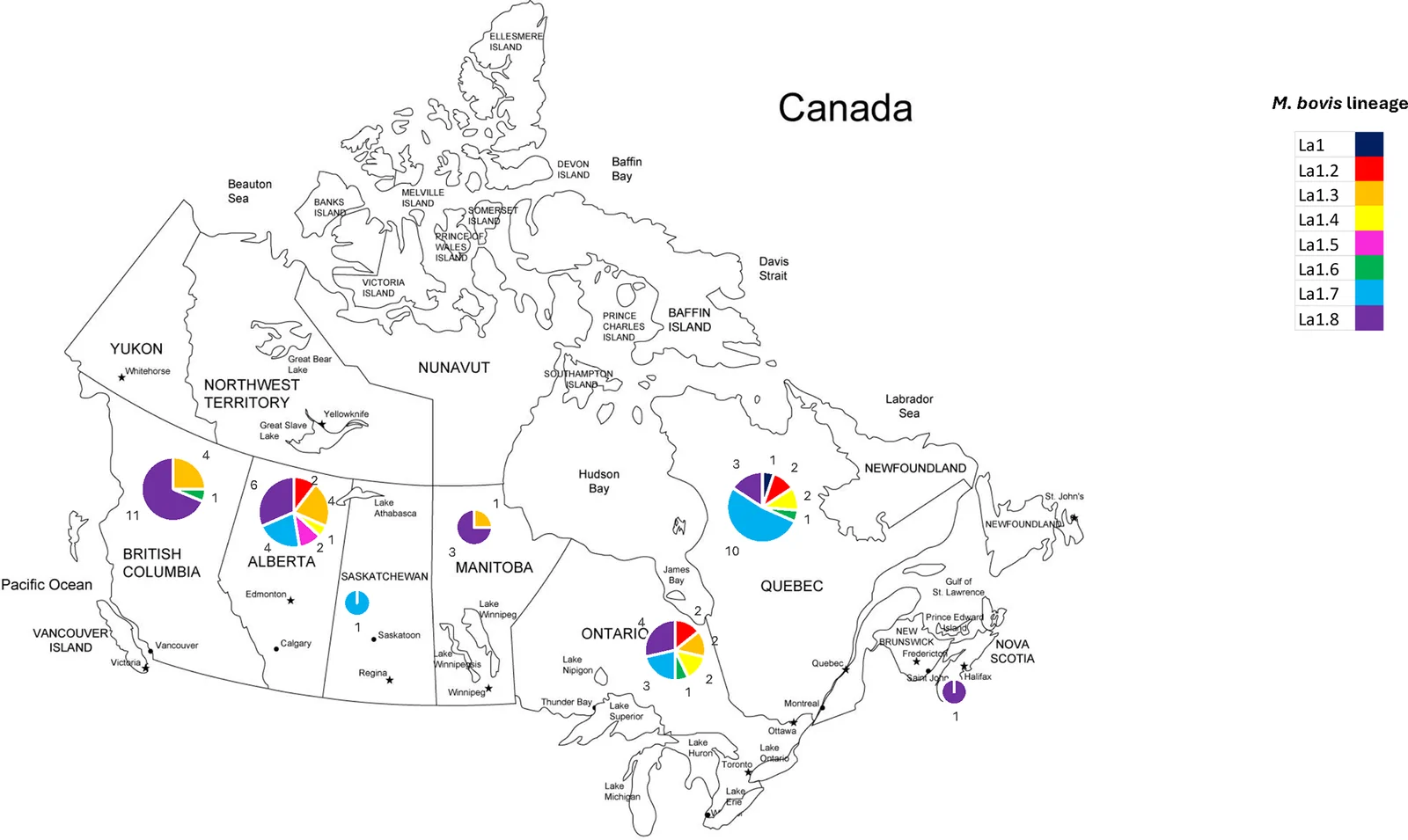
Geographic distribution of *M. bovis* La1 sublineages in Canada. Pie charts represent the proportion of sublineages in each province. Frequency of various *M. bovis* lineages and their geographical distribution by Canadian provinces. The size of the pie chart in a province is relative to the number of cases seen in the province, and the colors within the pie represent different La1 sublineages that are characteristic of *M. bovis*. Map of Canada data/image © Bruce Jones Design Inc. (2009). Royalty-free.

**Fig 2:**
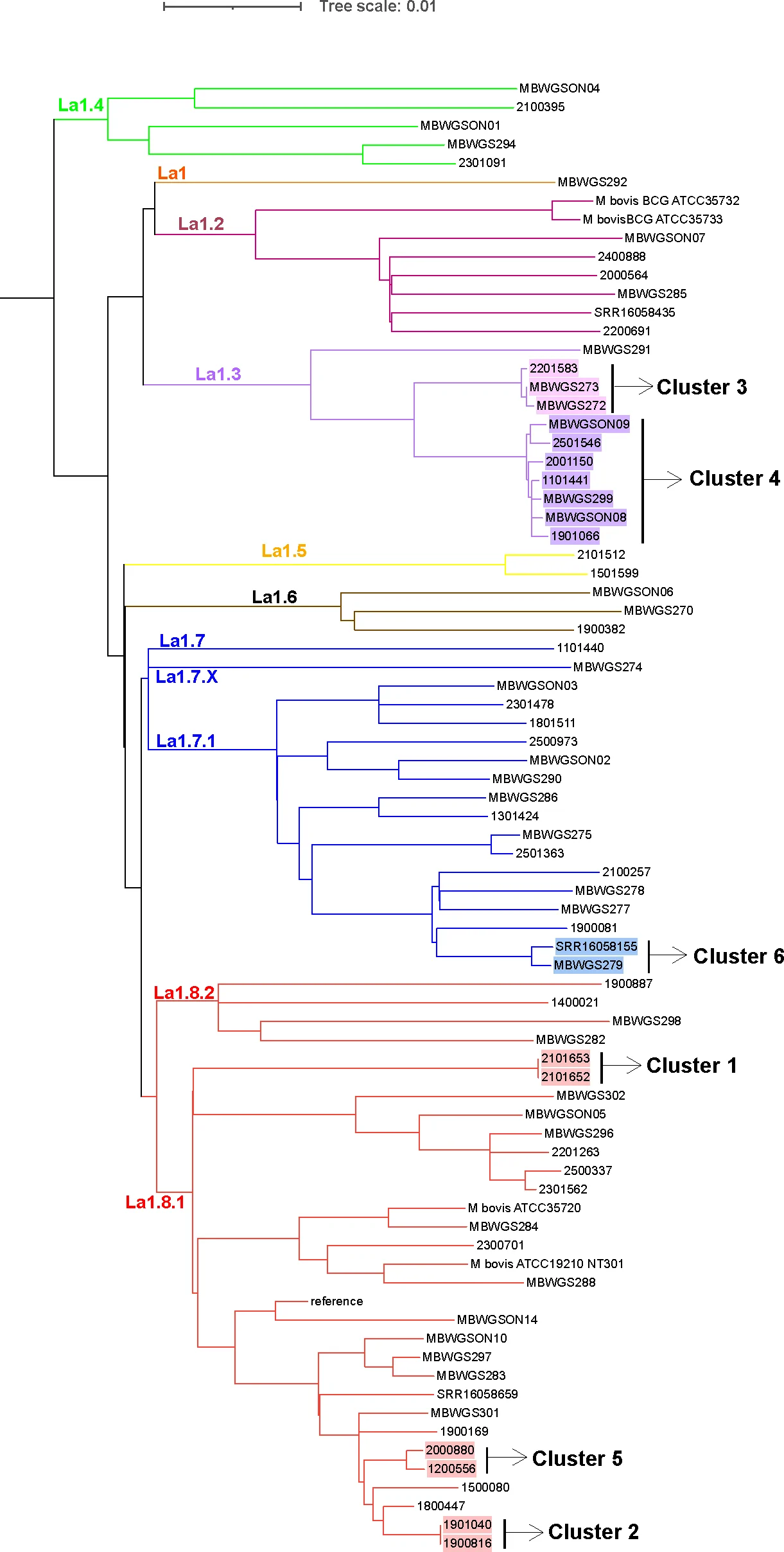
A phylogenetic tree was generated by SNVPhyl v 1.2.3 pipeline in IRIDA and visualized using iTOL version 7.5.1. Study isolates with a difference of 25 single nucleotide variants or fewer are shown with their respective cluster numbers, emphasizing samples that may exhibit potential relatedness. The lineage identification from La1 to La8 for each isolate and branch is depicted in various colors within the phylogenetic tree.

The sequence quality report indicated an average genome coverage of 127× for the study samples, with an average of 2,716,895 mapped reads and an average percentage of mapped reads at 96.7% relative to the *M. bovis* reference genome. All 74 clinical isolates and 4 ATCC strains belonged to animal lineage La1 with the specific sublineages identified for all samples except for one as this sample did not identify k-mers for sublineages. The total counts of the 74 isolates corresponding to sublineages were La1—1 (1.3%), La1.2—6 (8.1%), La1.3—11 (14.9%), La1.4—5 (6.8%), La1.5—2 (2.7%), La1.6—3 (4.1%), La1.7—18 (24.3%), and La1.8—28 (37.8%). The distribution of sublineages in each province is shown in Fig. 1. Among these, 24 out of 28 La1.8 were categorized as clonal complex (CC) Eu1 (La1.8.1), 16 out of 18 isolates from sub-lineage 1.7 were CC Eu2, 6 isolates genotyped as La1.2 were CC Eu3, and 11 isolates from sub-lineage La1.3 belonged to the CC African Af2, as shown in Fig. 2.

A summary of the phenotypic DST results for 72 of the *M. bovis* clinical isolates is described in Tables 3 and 4 with detailed data shown in Table S1. Data for first-line DST were not available for two isolates. None of the isolates exhibited phenotypic resistance to ethambutol; however, two isolates (2.8%) were found to be phenotypically resistant to isoniazid, and one isolate (1.4%) was resistant to rifampin. Genotypic resistance prediction was 94.4% (68/72) concordant with phenotypic DST results for isoniazid (Table 4) as two additional isolates demonstrating phenotypic resistance to isoniazid did not have any known mutation associated with resistance (Table 3). The single isolate with phenotypic rifampin resistance had a high-confidence resistance mutation in *rpoB* (p.Ser450Leu) detected. As expected, all isolates harbored the *pncA* p.His57Asp mutation, characteristic of *M. bovis* pyrazinamide resistance (41).

**TABLE 3:** Comparison of drug susceptibility profiles for *M. bovis* isolates with phenotypic resistance or mutations associated with isoniazid, rifampin, and ethambutol resistance

Isolate number	Phenotypic drug susceptibility result			Gene mutations TB-Profiler
	Isoniazid	Rifampin	Ethambutol	
MBWGS282	R	S	S	None
2101512	S	S	S	*ahpC* p.Pro2Ser
1501599	S	S	S	*ahpC* p.Pro2Ser
2501363	R	S	S	None
MBWGS272	S	R	S	*rpoB* p.Ser450Leu

**TABLE 4:** Summary of phenotypic drug susceptibility results and concordance with genotypic drug resistance prediction for *M. bovis* isolates identified across seven Canadian provinces, 2000–2025^*a*^

	DST susceptible	DST resistant	DST and TB-Profiler concordance
Isoniazid	70	2	94.4%
Rifampin	71	1	100%
Pyrazinamide	0	60	100%
Ethambutol	72	0	100%

^*a*^ DST, Phenotypic drug susceptibility testing results were available for 72 isolates for isoniazid, rifampin, and ethambutol and for 60 isolates for pyrazinamide.

The phylogenetic tree generated with SNVPhyl and *M. bovis* AF2122/97 as the reference strain included positions from 89.3% of the core genome for all samples. The SNVPhyl tree with cluster numbers and specific lineage and matrix are shown in Fig. 2 and Table S2. Samples with pairwise differences ranging from 0 to 25 SNVs were selected for in-depth analyses as a recommended SNV cutoff for this organism does not exist for humans, resulting in six potential clusters.

The cluster 1 was comprised of two isolates, 2101652 (tracheal aspirate) and 2101653 (lung fluid), which had no SNV difference and were from British Columbia. The cluster 2 also was comprised of two isolates from British Columbia; 1900816 (cerebrospinal fluid) and 1901040 (brain biopsy) had no SNV difference between the isolates. The cluster 3 was comprised of three isolates from British Columbia, 2201583, MBWGS272, and MBWGS273, all isolated from extrapulmonary sources, which had an SNV difference of 4–10 among them.

The cluster 4 was of seven isolates (three from Alberta, one from British Columbia, two from Ontario, one from Manitoba), 2501546, 1901066, MBWGSON09, 2001150, MBWGSON08, MBWGS299, 1101441, had a SNV difference of 9–25 between isolates, five of these strains were isolated from extrapulmonary sites, and two from unknown source (Table S1). The cluster 5 had two isolates from Briitsh Columbia with a 19 SNV difference between isolates 1200556 (sputum) and 2000880 (respiratory liquid aspirate). The cluster 6 was comprised of two isolates from Ontario and Quebec, SRR16058155 (NA) and MBWGS279 (lymph node biopsy), respectively, and had a SNV difference of 23.

## DISCUSSION

In Canada, human cases related to *M. bovis* are nationally reported as *M. tuberculosis* complex, but this is not the case for some countries despite it being a cause of TB as per the most recent zTB in human statistics from the 2020 Report (19). Briefly, in 2019, the WHO regions reported 140,000 cases of zTB in humans and 11,400 deaths worldwide (17). In some studies, the prevalence of *M. bovis* infections in human has ranged from as low as 0.4% to 76% in Tunisia (42, 43) to between 1% and 28% in Mexico (44, 45). *M. bovis* was found in 1.4% of human TB cases reported in 1995–2005 in the USA (46) but accounted for 11% of human cases in San Diego, California (47). Although, not every *M. bovis* isolated from humans was received for inclusion in our study. The number of collected isolates over the past decade has fluctuated between 1 and 7 annually (<1%), suggesting that the incidence of *M. bovis* (not BCG) among humans in Canada is likely low. These statistics are similar to those seen in the USA, with less than 2% identified annually (48, 49). Overall provincial distribution of *M. bovis* cases varied greatly, with higher absolute number of cases identified in the provinces of British Columbia, Alberta, Ontario, and Quebec. In the last 10 years, these provinces have seen between 10 and 18 cases each. Unfortunately, the data were not available to explore factors that may have influenced the number of cases at each provincial level. In 2024 Alberta Public Health investigation determined that all *M. bovis* cases in the province were imported by immigration or travel of young non-vaccinated individuals to regions where *M. bovis* is endemic (50).

The age of participants at the time of infection in our study sample varied from less than 1 year to as much as 90 years, with the highest number of cases occurring among individuals aged between 21 and 40 years. These statistics are different than what was seen in France where the elderly were more affected than younger individuals, likely due to stronger control measures in France to zTB in animals (51). However, our previous study supports the current findings where median age varied from 15 to 44 years for the majority of *M. bovis* cases between 1995 and 2020 (50).

In another study of 670 *M. bovis* isolates, 230 (34%) were derived from respiratory samples and 440 (66%) were originated from non-respiratory samples (10). Similarly, Riopel et al. (50) reported a higher portion of extrapulmonary *M. bovis* cases than pulmonary disease in Western Canada. Consistent with these findings, our data also showed that a greater proportion of isolates (60%) were obtained from non-respiratory specimens. This distribution likely reflects the predominant route of infection, consumption of unpasteurized dairy products, which is associated with a higher frequency of extrapulmonary disease compared to *M. tuberculosis*. These trends vary across major WHO regions due to differences in endemicity, geographic factors, and population-level exposure risks.

Although pyrazinamide phenotypic drug testing results were unavailable for all isolates, WGS-AMR predictions were available for all samples and identified the His57Asp mutation in the *pncA* gene in all study isolates indicating intrinsic resistance to pyrazinamide. Rifampin phenotypic and genotypic testing results were concordant for the 71 rifampin-sensitive and 1 rifampin-resistant isolate. The resistant isolate had a point mutation Ser450Leu in the *rpoB* gene, which is known to confer resistance to rifampin (12, 52). Out of 74 isolates, 72 isolates showed phenotypic sensitivity to ethambutol, and 2 did not have phenotypic DST results available. No mutation was seen for ethambutol. Data for isoniazid showed phenotypic resistance for two isolates that did not have a mutation in *inhA, katG,* or *ahpC* genes as per TB-Profiler v6.6.5. However, two isolates exhibited an *ahpC* p.Pro2Ser mutation and isoniazid-resistance prediction according to TB-Profiler, but DST results showed sensitivity; however, this mutation is not yet included in the WHO catalog (35, 53). This gene encodes for an enzyme involved in protecting cells from oxidative stress and a recent study found this mutation in *M. bovis* isolates and implied resistance to isoniazid (54). Additionally, *M. bovis* BCG ATCC35733 belonging to La1.2.BCG had a mutation *mmaA3* p.Gly98Asp, which is a known mutation for isoniazid resistance in BCG but not yet included in the WHO catalog of mutations (55).

Even though not all isolates could be obtained for testing, the number of isolates in the last 10 years suggests a low prevalence of *M. bovis* isolated from humans in Canada. The majority of isolates belonged to lineages La1.8 (37.8%), La1.7 (24.3%), and La1.3 (14.9%). As per current international nomenclature, CC seen in this study were Eu1, Eu2, Eu3, and Af2 (5). La1.7 was seen in four provinces (Alberta, Ontario, Quebec, and Saskatchewan), and La1.8 was seen in all provinces except SK. It is essential to identify species and lineage for all members of the MTBC in humans, as it helps in diagnosing infections, AMR, public health pathogen surveillance, and provide context to evolutionary relationship of strains and their lineage.

Many countries such as Canada, USA, Australia, New Zealand have effectively controlled bovine TB, while in certain nations, it continues to pose a considerable challenge to both animal and human health (1, 7, 16, 39, 41, 43, 52). Human transmission has been associated with the intake of unpasteurized milk but has not been linked to any significant outbreaks. Phylogenetic analysis of the study isolates shows clustering; however, the isolates may not be related as MTBC has exhibited a relatively low level of genetic diversity throughout time. This poses challenges in distinguishing genuine transmission events from coincidental infections solely based on genomic data, as the limited variation might create the illusion of a tightly-knit outbreak. The cluster 1 with two isolates originated from British Columbia and exhibited zero SNVs. Upon further investigation, it was concluded that they represented the same patient, and the isolates were collected 2 months apart and hence show close relatedness. Another two samples in cluster 2 from BC had 0 SNV, and it was found two different samples were submitted for a single case due to clinical reasons, and hence, the WGS showed similarity between the two. Cluster 3 had three isolates: MBWGS272 and MBWGS273 originated from BC and were isolated in 2008, while 2201583 was isolated in 2022 in the same region. These three samples exhibit SNV differences ranging from 4 to 10 SNVs. Accessing data for the first two strains with four SNV difference is challenging as these are old samples, and these two BC samples may be related; the third sample in the cluster, which is from 2022, is separated by a significant time interval and 10 SNV difference, suggesting it may not be related to the other two isolates.

The cluster 4 consisted of seven samples with a difference of 9–25 SNVs for three isolates from Alberta, one from British Columbia, two from Ontario, and one from Manitoba. Alberta isolates are from different years (2011, 2020, and 2025) and SNV > 6, suggesting unrelatedness, and hence, these are less likely to be a part of a recent outbreak. Cluster 5 with two isolates, 1200556 (AB) and 2000880 (BC), exhibited a difference of 19 SNVs between them and may not be linked with one another. Furthermore, they were isolated in different years and originated from different provinces, suggesting that they are likely unrelated. The cluster 6 consisted of two isolates and had a SNV difference of 23, and they were both from different provinces, Ontario and Quebec, and hence are likely unrelated.

Intra-species transmission of *M. bovis* within households has been observed. The infection of *M. bovis* in domestic cats indicates a transmission route between companion animals and humans, suggesting a potential risk (56). Other reported examples of *M. bovis* intra- and inter-species transmission include from deer to hunters in the USA, from cattle to a human and back to cattle in Switzerland (57), from cattle to human and human to human and pet in the same household in the UK (58), illustrating the importance of all contact tracing during the zTB investigations. This emphasizes the significance of a one-health approach by both animal and human healthcare professionals to address outbreaks and avert further transmission to humans, as well as to domestic and wildlife animals. The introduction of the bovine TB eradication program in Canada, almost one century ago, has almost eliminated the disease from agricultural livestock and brought the prevalence rate in cattle herds to less than 0.01% (59). The WGS analysis of representative *M. bovis* strains associated with bovine TB clusters reported in Canada during the last four decades did not reveal any transmission events between Canadian animal and human populations (Olga Andrievskaia, personal communication). It suggests the external origin of the human *M. bovis* infection in Canada. Integration of surveillance systems to better assess relatedness of strains derived from human and animal TB infections has value globally. A collaboration between animal and health sectors and partners should be initiated or maintained for better surveillance and control of zTB pathogens in humans and animals.

### Conclusions

*M. bovis* infections in humans are significant and must be monitored. Testing should include phenotypic DST, WGS-based AMR predictions, or any other molecular tests that are validated for clinical use. Establishing a national genomic database for zTB originating from both humans and animals is essential for tracking transmission. The origin of *M. bovis* is a critical factor, as importation from countries with endemic *M. bovis* infections is likely a primary reason for the presence of these strains in Canada. Furthermore, exchange and comparative analyses of animal and human *M. bovis* databases should be conducted to ensure harmonization of nomenclature, classification standards, and to support epidemiological investigations.

## Data Availability

Data belong to several government, research, and diagnostic laboratories, various agencies, and departments, public health programs, and hospitals. Due to issues with intellectual property rules for these participating organizations, the WGS data cannot be shared publicly at this time. Table S1 has been provided, containing line metadata and results from the bioinformatic pipelines.

## SUPPLEMENTAL MATERIAL

Supplemental legends spectrum.01485-26-s0001.docx Legends for Tables S1 and S2.

Table S1 spectrum.01485-26-s0002.xlsx Line data for study isolates with year of isolation, geographical information, sample category, phenotypic DST data along with BioHansel and TB Profiler results.

Table S2 spectrum.01485-26-s0003.xlsx Matrix of single nucleotide variants between isolates generated with SNVPhyl v 1.2.3. Isolates with {less than or equal to}25 SNV difference between them are indicated by green boxes outlined in a black box.

ASM does not own the copyrights to Supplemental Material that may be linked to, or accessed through, an article. The authors have granted ASM a non-exclusive, world-wide license to publish the Supplemental Material files. Please contact the corresponding author directly for reuse.
