# Supplementary material for: Genomic diversity of human-associated Mycobacterium bovis in Canada
Source: Microbiol Spectr. 2026 Oct 13;14(11):e01485-26. doi: 10.1128/spectrum.01485-26 (PMC13729206; doi:10.1128/spectrum.01485-26)
Supplement: Supplementary file 1 [file spectrum.01485-26-s0001.docx]

**Supplementary Table 1**

Line data for study isolates with year of isolation, geographical information, sample category, phenotypic DST data along with BioHansel and TB Profiler results.

**Supplementary Table 2**

Matrix of single nucleotide variants between isolates generated with SNVPhyl v 1.2.3. Isolates with ≤25 SNV difference between them are indicated by green boxes outlined in a black box.
